# Light involved regulation of BZR1 stability and phosphorylation status to coordinate plant growth in *Arabidopsis*

**DOI:** 10.1042/BSR20170069

**Published:** 2017-04-28

**Authors:** Qian-Feng Li, Li-Chun Huang, Ke Wei, Jia-Wen Yu, Chang-Quan Zhang, Qiao-Quan Liu

**Affiliations:** Key Laboratory of Crop Genetics and Physiology of Jiangsu Province/Key Laboratory of Plant Functional Genomics of the Ministry of Education/Co-Innovation Center for Modern Production Technology of Grain Crops, College of Agriculture, Yangzhou University, Yangzhou 225009, China

**Keywords:** gene expression and regulation, protein stability, plant hromones, post translational modification

## Abstract

Light and brassinosteroid (BR) are master environmental stimulus and endogenous cue for plant growth and development respectively. Great progress has been made in elucidating the molecular mechanisms on the cross-talk between light and BR. However, little is known about how BZR1, the pivotal integration node, is regulated by light and dark. Here, we demonstrated that an intact BR signaling pathway is essential for dark-induced hypocotyl elongation. Consequent expression assay showed that light–dark switch affected BZR1 phosphorylation and accumulation. Moreover, blocking the 26S proteasome pathway promoted the accumulation of both phosphorylated and dephosphorylated BZR1 proteins. Restriction of new protein biosynthesis had multiple effects on BZR1 phosphorylation status and stability, relying on the availability of light and the 26S proteasome pathways. Furthermore, sugar treatment strikingly enhanced the accumulation of total BZR1 under either light or dark conditions, likely by repressing transcript abundance of *MAX2*, a gene encoding an E3 ligase for BZR1. Finally, light-regulated phosphorylation change of BZR1 requires the existence of endogenous BR as well as functional BIN2 and protein phosphatase 2A (PP2A). Taken together, our results depicted a light-involved complex regulation network of BZR1 stability and phosphorylation status.

## Introduction

Plants are sessile organisms and their growth and development are controlled by multiple environmental cues and endogenous hormones. Light, the energy source for plant photosynthesis, is also one of the most important environmental factors for normal plant growth and development, such as seed germination and seedling photomorphogenesis etc. For example, seedlings that germinated in the dark exhibited elongated hypocotyls, small and folded cotyledons with undifferentiated chloroplasts, and repression of light-induced genes. Whereas the exposure of seedlings to light causes a developmental switch from skotomorphogenesis to photomorphogenesis, characterized by short hypocotyls, opened and expanded cotyledons, and differentiation of chloroplast. How can light trigger such a dramatic morphology change of plant? One of the most important reasons is that light could regulate thousands of target genes related to plant growth and development via the light signaling pathway. Several classes of transcription factors, including the bHLH proteins phytochrome interacting factors (PIFs) [1] and the b-ZIP protein elongated hypocotyl 5 (HY5) [2] could directly bind to the G-box of their target genes and therefore modulate their expression to mediate light responses [3]. Moreover, light could also orchestrate plant architecture via interacting with other pathways, including multiple hormonal pathways. For instance, growth promoting hormone brassinosteroids (BRs) are involved in light-regulated plant photomorphogenesis. Dark-grown BR deficient or BR insensitive mutant exhibits de-etiolation phenotypes, including shortened hypocotyls, opened cotyledons, and elevated expression of many light-induced genes [4–7].

BRs, a group of steroidal hormones in plants, are known mainly for promoting organ growth and regulating a broad spectrum of plant developmental and physiological responses. Recently, combined genetic and biochemical approaches have greatly advanced our understanding of BR signal transduction pathway in *Arabidopsis* [7–9]. Generally, BR directly binds to the extracellular domain of the transmembrane leucine-rich repeat containing receptor-like kinase (LRR-RLK) BRI1 [10], which results in fast activation of BRI1’s intracellular kinase domain. The activated BRI1 triggers a series of downstream phosphorylation events and subsequently inactivates the GSK3/Shaggy-like protein kinase BIN2, a pivotal negative regulator of BR signaling [11–15]. Consequently, BZR1 and BES1, two closely related transcription factors, are rapidly dephosphorylated by protein phosphatase 2A (PP2A) [16]. The dephosphorylated BZR1 and BES1 could move into the nucleus and bind the E-box and BRRE elements of their target genes [15,17–19]. When BR level is low, BIN2 could phosphorylate and inactivate BZR1 and BES1 by modulating their cytoplasm-retention and DNA-binding activity [7].

Although the interaction between light and BR has long been observed [5,6,20,21], the underlying molecular mechanisms were only started to be understood recently. Luo et al. [22] showed that the transcription factor BZR1 in the BR pathway could directly interact with GATA2 in the light signaling pathway to regulate hypocotyl elongation of seedlings. Another proposed model depends on the interplay between BZR1 and PIF4, a key transcription factor in light signaling [23]. BZR1 directly interacts with PIF4 to co-regulate approximately 2000 common target genes, a large number of which were synergistically regulated by BZR1 and PIF4. The study proposed a model that the BZR1–PIF4 heterodimer co-regulate a core transcription network to coordinate plant growth in response to endogenous and environmental stimuli. In addition, direct interactions were also reported between BZR1 and two E3 ubiquitin ligases, COP1 and MAX2, shedding some light on the degradation mechanisms of BZR1 [24,25]. More recently, a direct interaction between BZR1 and HY5 was also identified in coordinating the cotyledon opening of dark-grown seedlings [26]. These studies not only depicted a complicated integration network between BR and light signaling pathways, but also highlighted the central roles of BZR1, an integration node of the signaling cross-talk, in coordinating plant growth and development. It is well known that BR could affect BZR1 activity by modulating its phosphorylation status, thus altering BZR1’s cytoplasm-retention and DNA-binding activity [11,13,15,18]. Moreover, modulation of BZR1 stability is another critical element to affect BR signaling output [24,26,27]. Although the above studies have successfully uncovered direct connections between BZR1 and multiple components in the light signaling pathway, how BZR1 itself, the principal signaling integrator, is regulated under both light and dark conditions has not been studied in detail yet.

The pivotal roles of BZR1 in both BR and light signaling pathways promoted us to investigate how BZR1 stability and activity were regulated by some other pathways, especially light. In the course of the present study, we discovered that light–dark switch could alter the phosphorylation status and abundance of BZR1, nevertheless, requiring the availability of an intact BR signaling pathway. Moreover, BZR1 accumulation and activity were also dependent on the 26S proteasome pathway, new protein biosynthesis and sugar levels. Thus, the present study shed some light on the regulation network of BZR1 protein, a key transcription factor in the BR signaling pathway.

## Materials and methods

### Plant materials and growth conditions

The BR mutants used in the present study included *bri1-5, bzr1-1D*, and *det2*, and their respective wild-types Wassilewskija-2 (WS) and Columbia (Col-0). Transgenic *Arabidopsis* lines were generated by the Agrobacterium-mediated floral dip method. Sterilized *Arabidopsis* seeds were grown on Murashige and Skoog (MS) medium with sucrose (15 g/l) under a 16 h/8 h light/dark cycle at 22°C. White light was supplied by cool-white fluorescent lamps (Philips TLD30W/865 tubes, 10 μmol m^–2^ s^–1^; Quantitherm Light Meter). For hypocotyl elongation experiments, 5-day-old seedlings were transferred to new plates only with phytoblend and kept in continuous light or darkness for another 2 days. To eliminate the endogenous BR, seeds were germinated and grown in MS medium supplied with BRZ (2 μM) or the mock for 6 days.

### Chemical treatment in both light and dark conditions

For light or dark treatment experiments, 7-day-old normal grown seedlings were transferred to ddH_2_O and treated in the light or dark for 20 h. For the time-course experiment, the seedlings were kept in the light or dark for 4, 8, and 20 h respectively. For the pharmacological treatment experiments, various chemicals were used in ddH_2_O for treatment of the seedlings in both light and dark conditions. Cycloheximide (CHX) (100 μM) (SIGMA) and MG132 (10 μM) (SIGMA) were used to treat the seedlings for 8 h to block BZR1 biosynthesis and degradation respectively. LiCl (10 mM) and Canth (10 μM) were used for inhibiting the activity of BIN2 and PP2A respectively. To test the effect of sugar on the accumulation of BZR1, 90 mM sucrose was added in ddH_2_O to treat seedlings for 20 h.

### Hypocotyl length measurement

Seven-day-old seedlings were collected and laid horizontally on an agar plate, photographed, and hypocotyl lengths were analyzed by using the software ImageJ. At least 20 seedlings were measured for each genotype in each set of experiments.

### RNA isolation and real-time quantitative RT-PCR assays

Total RNA was extracted using the RNeasy plant mini kit (Qiagen), treated with DNase I (Qiagen) and reverse transcribed by using the SuperScriptTM first-strand synthesis system (Invitrogen). Real-time quantitative RT-PCR (qRT-PCR) was performed by using the MyiQ real-time system (Bio-Rad) and the iQ SYBR-Green Supermix (Bio-Rad). *UBQ10* was used as internal control for sucrose-related and light-related experiments. All qRT-PCR reactions were performed in three technical replicates using total RNA samples extracted from three independent biological replicate samples. The PCR primers used are listed in Supplementary Table S1.

### Immunoblot analysis

Seedlings grown on MS plates were collected directly or treated with 1 μM BL for 2 h, or kept in the continuous light or dark conditions for 20 h. The entire seedlings of 10-day-old *Arabidopsis* plants were collected and ground into powder in liquid nitrogen. SDS sample buffer (2×) was added in the ratio of 1:1 (1 ml of buffer to 1 mg of tissue powder) to extract the proteins. The extracted proteins were then heated at 70°C for 10 min, followed by centrifugation at 12000 ***g*** for 10 min. The resulting supernatants were transferred to a new microfuge tube. SDS/PAGE was performed to resolve the protein extracts. After electrophoresis, proteins were transferred to a PVDF (polyvinylidene difluoride) membrane (Millipore) with a semi-dry electrophoretic transfer cell (Bio-Rad) and immunodetected with antibodies recognizing GFP (Clontech) or myc (Sigma).

## Results

### BR biosynthesis and signaling are necessary for dark-induced hypocotyl elongation in *Arabidopsis*

**Figure 1:**
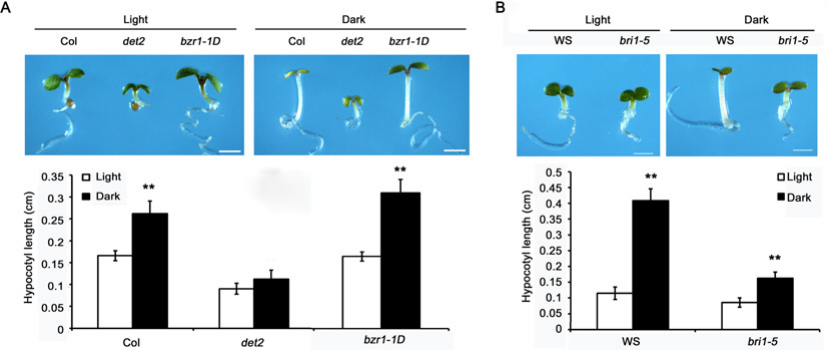
BR biosynthesis and signaling are necessary for dark-induced hypocotyl elongation in *Arabidopsis* (**A**) Five-day-old light-grown Col, *det2*, and *bzr1-1D* seedlings were treated with light and dark for another 2 days respectively. (**B**) Five-day-old *bri1-5* and WS seedlings were treated with light and dark for another 2 days respectively. Data are means ± SD (*n*>20). Three biological repeats were performed and asterisks indicate the levels of statistical significance as determined by Student’s *t* test; **P<0.01, scale bar = 0.1 cm.

It is well noted that light plays a critical role in the regulation of plant growth, and dark-grown *Arabidopsis* seedlings exhibit much longer hypocotyls than their light-grown counterparts [21]. Also, it has been reported that the hypocotyl elongation of dark-grown BR deficient or insensitive mutants are restrained [23]; however, how BR mutants respond to the stimulation of light–dark switch are still unclear. In the present study, 5-day old light-grown *Arabidopsis* seedlings, including BR mutants and their respective wild-type controls, were kept in light or dark for another 2 days. The result showed that the hypocotyl length of dark-treated Col is much longer than that of light-treated control, while the effect of dark-induced hypocotyl growth was strongly reduced in the BR deficient mutant *det2* (Figure 1A), indicating that the *det2* mutant is less sensitive to the dark-stimulated cell elongation. As to *bzr1-1D*, a dominant mutant of BZR1 was more sensitive to dark-induced hypocotyl elongation when compared with its wild-type control Col (Figure 1A). To further confirm this notion, the BR-insensitive mutant *bri1-5* was also used to test its growth response to dark treatment. The result showed that *bri1-5*, similar to the BR-deficient mutant *det2*, was also less sensitive to dark-induced cell elongation in *Arabidopsis* (Figure 1B). The above results suggested that both the existence of endogenous BR and an intact BR signaling pathway are required in the light–dark switch stimulated cell elongation in *Arabidopsis*.

### Light–dark switch could modulate the accumulation and phosphorylation status of BZR1

**Figure 2:**
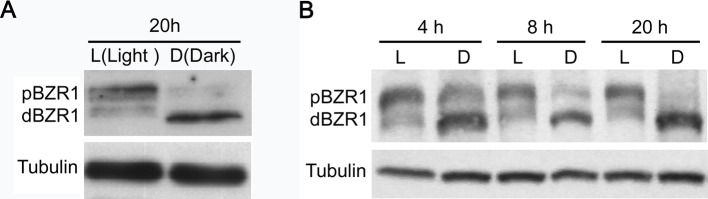
Dark treatment induces the dephosphorylation of BZR1 (**A**) Dark treatment of the W2C (*pBZR1::BZR1-CFP*) seedlings for 20 h remarkably induced the dephosphorylation of BZR1. (**B**) A time-course analysis of dark-induced BZR1 dephosphorylation in W2C. In (A) and (B), Tubulin was used as loading control; L, light; D, dark.

According to the results of previous studies, the master transcription factor BZR1 is supposed to function as the integration node of light and BR pathways [22–24,26]. Therefore, we examined the effect of light- and dark-treatment on the accumulation of BZR1 protein. The result showed that BZR1 exhibited a different protein abundance and phosphorylation status between light- and dark-treated samples (Figure 2A). To further investigate how light and dark modulate BZR1 protein, a detailed time-course analysis were carried out. The Western blot result showed that even a 4-h dark treatment remarkably induced the dephosphorylation of BZR1 when compared with that of the light-treated samples. As the extention of treatment time, the phosphorylated BZR1 was almost disappeared and the dephosphorylated BZR1 was accumulated in the dark-treated samples. As to the light-treated samples, little difference was observed (Figure 2B). The results suggested that light/dark treatment could modulate the stability and phosphorylation status of BZR1.

### The stability of BZR1, including the phosphorylated and dephosphorylated forms, was regulated by the 26S proteasome pathway in both light and dark conditions

**Figure 3:**
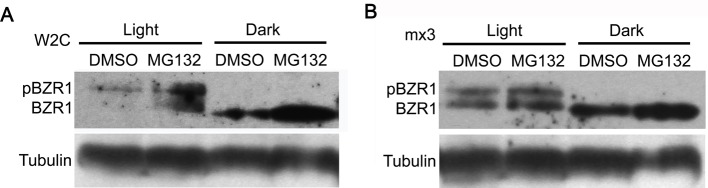
MG132 treatment inhibited the degradation of both pBZR1 and dBZR1 in either light or dark conditions (**A**) The abundance of total BZR1 (pBZR1 and dBZR1) was increased in the MG132-treated W2C seedlings in both light and dark conditions. (**B**) The abundance of total BZR1 (pBZR1 and dBZR1) was increased in the MG132-treated mx3 (*pBZR1::mBZR1-CFP*) seedlings in both light and dark conditions. *mBZR1* is the *BZR1* gene with the *bzr1-1D* mutation. The expression of BZR1 was detected by using an anti-GFP antibody. In (A) and (B), Tubulin was used as loading control.

Previous studies mentioned that the phosphorylated BZR1 was inactive and unstable, which was easily to be degraded by the 26S proteasome pathway [11]. To examine whether 26S proteasome pathway also functions in the light/dark regulated BZR1 stability, a number of experiments were performed. First, the seedlings of *pBZR1::BZR1-CFP* were treated with MG132, an inhibitor of 26S proteasome pathway, for 20 h in both light and dark conditions. The result of Western blot assay indicated that MG132 treatment indeed increased the amount of total BZR1 in light, including both phosphorylated and dephosphorylated BZR1 (Figure 3A). As to the dark-treated seedlings, only dBZR1 could be detected in both the MG132- and DMSO-treated samples. Moreover, much higher amount of dBZR1 protein could be detected in the MG132-treated seedlings than that in the DMSO-treated controls (Figure 3A). The experiment demonstrated that MG132 could inhibit the degradation of both phosphorylated and dephosphorylated BZR1 in either light or dark condition. To further confirm this notion, a similar experiment was done by using the *proBZR1::mBZR1-CFP* transgenic seedlings, harboring a mutant *BZR1* gene from the dominant *bzr1-1D* mutant. The result showed that MG132 treatment could increase the amount of total mBZR1 (including both phosphorylated and dephosphorylated forms) in the light and dephosphorylated mBZR1 in the dark (Figure 3B), consistent with the result by using *pBZR1::BZR1-CFP* seedlings. Generally, difference of mBZR1 protein abundance between MG132- and DMSO-treated samples was small, suggesting that mBZR1 is more stable than the native BZR1 protein, which might be an important reason why *bzr1-1D* mutant exhibited a continuous and strong BR response. Therefore, the above results demonstrated that the 26S proteasome pathway is the major factor that regulates the stability of both phosphorylated and dephosphorylated BZR1.

### Inhibiting 26S proteasome pathway could not recover the reduction of BZR1 protein abundance caused by overexpression of HY5

**Figure 4:**
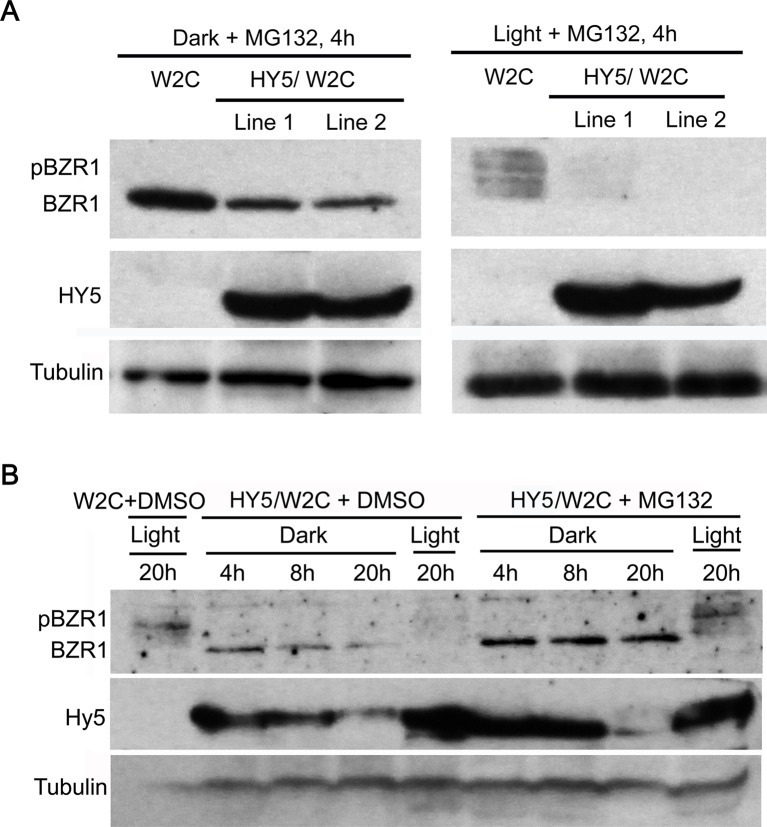
MG132 treatment could not recover the reduction of BZR1 protein abundance caused by overexpression of HY5 (**A**) Comparison of BZR1 protein abundance in response to MG132 treatment between the HY5 overexpression lines and the control in both dark and light conditions. (**B**) A detailed time-course analysis of the effect of MG132 on BZR1 protein abundance in the HY5 overexpression lines. An anti-GFP and an anti-MYC antibody were used to detect the expression of BZR1 and HY5 respectively. Tubulin was used as loading control.

Moreover, it was reported that overexpression of HY5, a primary transcription factor in the light signaling pathway, could obviously diminish the accumulation of BZR1 [26]. To examine whether MG132 treatment could recover the BZR1 protein abundance in the HY5 overexpression lines, we compared BZR1 protein abundance between *35S::HY5-Myc/pBZR1::BZR1-CFP* transgenic lines and *pBZR1::BZR1-CFP* controls in response to MG132 treatment. The Western blot result showed that the total BZR1 protein abundance was much less in the HY5 overexpression lines than that in the control seedlings under both light and dark conditions (Figure 4A), which suggested that HY5-induced reduction of BZR1 was not through the 26S proteasome pathway. Next, a time-course analysis was carried out to further study the accumulation of BZR1 protein regulated by dark and MG132 treatment. First, BZR1 protein abundance in the DMSO-treated *pBZR1::BZR1-CFP* seedlings was much higher than that in the HY5-overexpression lines in the light (Figure 4B, lane 1 versus lane 5), but it was similar to the MG132-treated HY5-overexpression plants (Figure 4B, lane 1 versus lane 9). Second, BZR1 was dephosphorylated in response to dark treatment in the DMSO-treated HY5-OE lines, but the total amount of BZR1 was decreased as the extension of treatment time. Third, the MG132-treated HY5-OE lines exhibited dBZR1 in the dark but its protein abundance did not alter even after 20 h’s incubation. The above results indicated that the 26S proteasome pathway controls the degradation of both pBZR1 and dBZR1, but it is not responsible for the HY5-induced reduction of BZR1 protein.

### The homeostasis of BZR1 stability and phosphorylation status are affected by BZR1 biosynthesis and degradation under both light and dark conditions

**Figure 5:**
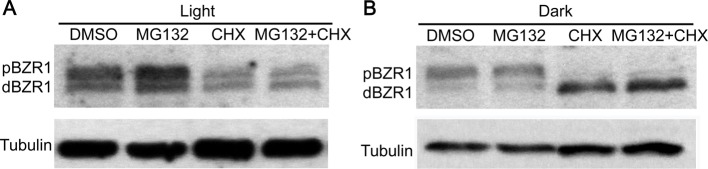
Blocking BZR1 biosynthesis and degradation affected the homeostasis of BZR1 stability and phosphorylation status (**A**) The effect of CHX and MG132 treatment on the BZR1 abundance and phosphorylation status in the light. (**B**) The effect of CHX and MG132 treatment on the BZR1 abundance and phosphorylation status in the dark. In (A) and (B), *pBZR1::mBZR1-CFP* seedlings were used. Tubulin was used as loading control.

The above findings, in combination with previous work [11,24], suggested that the 26S proteasome-mediated degradation of BZR1 played a pivotal role in modulating BZR1 protein abundance under both light and dark conditions. To clarify whether BZR1 biosynthesis is also involved in light-regulated BZR1 accumulation, a pharmacological experiment was performed to block new protein biosynthesis by treating the *pBZR1::mBZR1-CFP* transgenic seedlings with CHX (an inhibitor of protein synthesis). The expression result indicated that CHX treatment notably reduced while MG132 treatment slightly increased the total BZR1 protein abundance in comparison with the DMSO-treated controls in the light (Figure 5A). However, when MG132 and CHX were treated together, the accumulation of total BZR1 is just similar to that of CHX-treated alone (Figure 5A), implying that the increased BZR1 abundance in MG132-treated samples might rely on BZR1 biosynthesis. To find out what will happen in the dark, similar pharmacological treatment was carried out in darkness. The result clearly showed that once BZR1 biosynthesis was blocked by CHX treatment, the darkness would accelerate the dephosphorylation of BZR1 and led to a much higher accumulation of dBZR1 protein than that in the DMSO-treated controls (Figure 5B). The treatment with CHX and MG132 together had a similar effect on BZR1 protein abundance and phosphorylation status as CHX-treated alone (Figure 5B). All these data demonstrated that BZR1 mainly accumulates in the dephosphorylated form, indicating that BZR1 accumulation *in vivo* was a dynamic balance of its biosynthesis and degradation. If there is no new protein synthesized, the degradation mechanism would be attenuated or blocked. This result would shed light on our understanding of the regulation of BZR1 homeostasis *in vivo*.

### Dark-induced BZR1 dephosphorylation was independent of sucrose treatment

**Figure 6:**
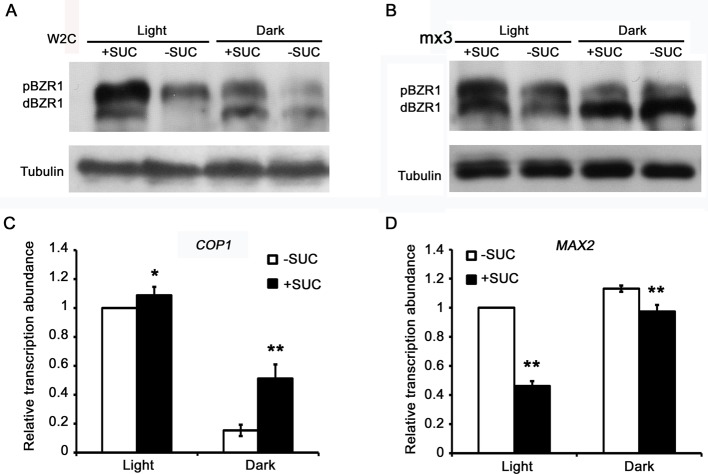
Sucrose treatment remarkably increased BZR1 protein abundance and modulated the transcription of two E3 ligases for BZR1 in both light and dark conditions (**A**) Sucrose induced the accumulation of both pBZR1 and dBZR1 in *pBZR1::BZR1-CFP* transgenic seedlings. (**B**) Sucrose increased the abundance of both pBZR1 and dBZR1 in *pBZR1::mBZR1-CFP* transgenic seedlings. In (A) and (B), tubulin was used as loading control. (**C**) The expression of *COP1* in response to sucrose treatment. (**D**) The expression of *MAX2* in response to sucrose treatment. In (C) and (D), RNAs were extracted from 5-day-old light-grown Col seedlings treated with 90 mM Suc for 20 h in both light and dark conditions. *UBQ10* was used as the internal control. Each value is the mean of three biological replicates. Asterisks indicate the levels of statistical significance as determined by Student’s *t* test; *P<0.05, **P<0.01.

Recently, it was reported that sugar, the product of photosynthesis and also an important environmental stimulus, not only interacts with BR pathway but also affects accumulation of BZR1 protein [28–30]. Therefore, it is essential to clarify how sugar and light co-regulate BZR1 stability and phosphorylation status. By treating the *pBZR1::BZR1-CFP* seedlings with sucrose, it was shown that sucrose treatment remarkably increased the total protein abundance of BZR1 in either light or dark conditions when compared with the non-treated controls respectively (Figure 6A). In addition, with the existence of sucrose, the light-treated seedlings accumulated much more pBZR1 than that in the dark-treated samples (Figure 6A). Without sucrose treatment, a similar conclusion could be drawn by comparing the light- and dark-treated samples. Furthermore, the *ProBZR1::mBZR1-CFP* seedlings were also used for testing and a similar result was obtained, such as the dark-induced dephosphorylation of BZR1 (Figure 6B). However, certain differences still existed. For instance, the ratio of dBZR1 to pBZR1 was much higher and the difference between sucrose treated and non-treated samples was smaller (Figure 6B). Therefore, it could be concluded that dark-induced BZR1 dephosphorylation was independent of sucrose levels. It is possible that sucrose improved BZR1 accumulation by suppressing its degradation, possibly via decreasing the expression of its E3 ligases. To test this hypothesis, transcription of *COP1* and *MAX2*, two genes encoding recently reported E3 ubiquitin ligases responsible for BZR1 degradation, was assessed by using qRT-PCR. The result showed that sucrose slightly increased the transcript abundance of *COP1* in both light and dark conditions (Figure 6C). As to *MAX2*, sucrose significantly repressed its transcript accumulation in the light and slightly reduced its transcript abundance in the dark (Figure 6D), which was consistent with the result of Western blot analysis (Figure 6A and B). The qRT-PCR result implied that *MAX2* might mediate sucrose-induced BZR1 accumulation in both light and dark conditions.

### Endogenous BR, functional BIN2 and PP2A were essential for the light–dark switch-induced phosphorylation change of BZR1

**Figure 7:**
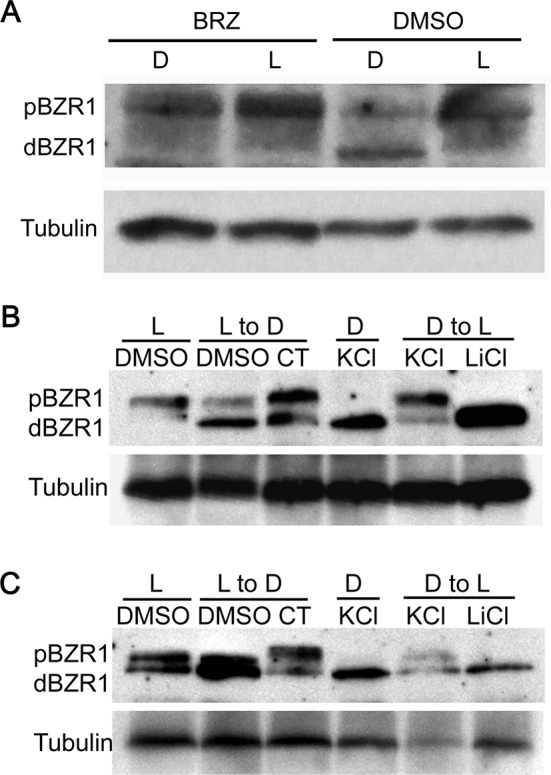
Light-regulated phosphorylation change of BZR1 requires the existence of endogenous BR as well as functional BIN2 and PP2A (**A**) Dark could not induce BZR1 dephosphorylation in the absence of endogenous BR. To eliminate the endogenous BR, seeds were germinated and grown in MS medium supplied with BRZ (2 µM) or its mock under a 16 h/8 h light/dark cycle for 6 days. Then, the 6-day-old seedlings were treated with light or dark for another 20 h. (**B** and **C**) CT treatment alleviated the dark-induced BZR1 dephosphorylation and LiCl treatment abolished the light-induced BZR1 phosphorylation in the *pBZR1::BZR1-CFP* seedlings (B) and *pBZR1::mBZR1-CFP* seedlings (C) respectively. Tubulin was used as loading control in (A), (B), and (C).

It is well established that BR could modulate the dephosphorylation and phosphorylation of BZR1 via phosphatase PP2A and kinase BIN2. To uncover whether the light–dark switch triggered phosphorylation change of BZR1 was dependent on the BR pathway, *pBZR1::BZR1-CFP* transgenic seedlings were grown in the medium supplemented with BRZ to deplete the endogenous BR. The result showed that dark-induced BZR1 dephosphorylation was abolished when the endogenous BR was absent (Figure 7A). Meanwhile, dark-induced dephosphorylation of BZR1 still existed in the DMSO-treated controls, suggesting that dark-induced BZR1 dephosphorylation requires the existence of endogenous BR. To further clarify whether the light–dark switch-modulated BZR1 phosphorylation change was mediated by PP2A and BIN2, the phosphatase and protein kinase in the BR signaling pathway, the specific inhibitors of PP2A and BIN2, were applied to seedlings respectively. The result showed that the treatment of *pBZR1:BZR1-CFP* with cantharidin (CT), the specific inhibitor of PP2A, significantly blocked the dark-induced dephosphorylation of BZR1 (Figure 7B, lane 2 versus lane 3), suggesting that PP2A played an important role in this process. Moreover, we used a specific GSK3 kinase inhibitor, lithium chloride (LiCl), to treat the *pBZR1:BZR1-CFP* seedlings and the result indicated that LiCl absolutely suppressed the phosphorylation of BZR1 in response to light treatment, whereas the KCl-treated control was not affected (Figure 7B, lane 6 versus lane 5). The same treatment was also applied to the *pBZR1:mBZR1-CFP* seedlings, in which a similar result was got (Figure 7C). Therefore, the above experiments demonstrated that an intact and active BR signaling pathway was essential for the light/dark-regulated phosphorylation change of BZR1, which was directly mediated by PP2A and BIN2.

## Discussion

Light and BR are master environmental stimulus and endogenous cue for plant growth and development respectively, and their interaction has been enthusiastically studied. To date, two distinct types of interaction mechanisms were revealed. One is that transcriptional regulation of the signaling components of one pathway by key components of the other pathway. For instance, BZR1 could suppress plant photomorphogenesis by directly modulating transcription of target genes. Generally, 14 of the total 16 light-signaling transcription factors are BZR1 direct targets [7]. The other mechanism relies on the direct protein–protein interaction between the key signaling components of the light and BR signaling pathways. The most well-known example is the interaction between BZR1 and PIF4 to mediate the cell elongation process during plant growth and photomorphogenesis [23,31,32]. Besides the direct interaction between key transcription factors, some other protein–protein interaction modules were also revealed. For instance, BR signaling kinase BIN2 could directly phosphorylates PIF4 to mediate its proteasome degradation [33]. Furthermore, increasing evidence revealed that multiple molecular mechanisms coexist on the BR-light signaling cross-talk. Do these different interaction modules function alone or work together? How do they coordinate diverse developmental and physiological processes spatially and temporally is another critical question to be answered in the future.

The studies mentioned above not only uncovered a complicated regulation network between BR and light, but also defined a central role of BZR1 in integrating BR and light pathways. As a pivotal integration node, BZR1 requires constantly altering its activity and stability to fulfill its role in various situations. It is well established that BZR1 activity is tightly correlated with its phosphorylation status, which is directly regulated by BR input. The phosphorylation states of BZR1 are currently considered to be reliable molecular gauges to assess the level of the BR signal output [11,13,14,34]. Such phosphorylation states modulate various functions of BZR1, including transactivation activity, DNA-binding affinity, protein stability, and spatial redistribution [35–37]. Studies indicated that phosphorylation induces the nuclear export of BZR1 via an interaction with 14-3-3 proteins [35–37]. Furthermore, BZR1 accumulation and stability are also modulated by multiple factors, including RGA, COP1, MAX2, HY5 etc [24–27]. For example, COP1, a dark-activated ubiquitin E3 ligase in the light signaling pathway, can also capture and degrade the inactive form of pBZR1, in this way to increase the ratio of active form of BZR1 and promote plant hypocotyl elongation [24].

Although the direct connections between BZR1 and multiple components in the light signaling pathway have been uncovered, how BZR1 itself is regulated by light has not been systematically studied yet. In the present study, we found that light–dark switch could obviously modulate BZR1 phosphorylation status, which requires the existence of endogenous BR. Further evidence demonstrated that PP2A- and BIN2-mediated light–dark switch induced BZR1 dephosphorylation and phosphorylation respectively. Together these data demonstrated that an intact and functional BR signaling pathway is required for light-regulated BZR1 phosphorylation. Moreover, we found that MG132 could elevate the accumulation of both pBZR1 and dBZR1 (Figure 3A and B). Previous reports showed that MG132 increased the accumulation of pBZR1 more than dBZR1 in light via blocking the 26S proteasome pathway [11]. We speculated that the divergence might come from the different treatment conditions, especially the presence of light or not. Our study demonstrated that light is an important regulator of BZR1 protein stability. It is possible that MG132 mainly enhanced pBZR1 abundance and then darkness could transfer the accumulated pBZR1 into dBZR1. In addition, the decreased BZR1 abundance caused by HY5 overexpression could not be rescued by MG132 treatment, implying a different regulation module existed. Furthermore, obstructing of BZR1 biosynthesis by CHX reinforced the dark-induced BZR1 dephosphorylation, implying that dBZR1 protein is more stable and easily to accumulate, especially in darkness. This result depicted a complicated and coordinated regulation of BZR1 abundance and phosphorylation status by multiple factors, including BZR1 biosynthesis inhibitor, BZR1 degradation inhibitor, and also the environmental stimuli. Finally, the result showed that dark-induced BZR1 dephosphorylation was independent of sucrose treatment, which could strikingly promote the accumulation of total BZR1 protein, possibly by suppressing the expression of E3 ligase MAX2.

**Figure 8:**
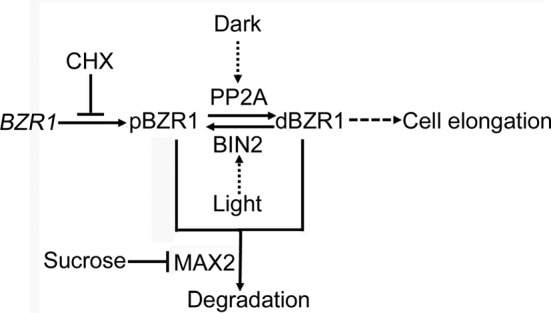
A proposed model illustrates the effects of light/dark, sucrose, and the 26S proteasome pathway on the stability and phosphorylation status of BZR1 Briefly, fresh synthesized BZR1 protein might be in the inactive phosphorylated form and CHX treatment could block BZR1 biosynthesis. Dark induces the dephosphorylation while light promotes the phosphorylation of BZR1, mediated by PP2A and BIN2 respectively. dBZR1, the active and stable form of BZR1, can activate the expression of downstream target genes and promote cell elongation. On the other hand, pBZR1, the unstable form of BZR1, is easily degraded by the 26S proteasome pathway, mediated by multiple E3 ubiquitin ligases. Finally, sucrose could significantly promote the accumulation of total BZR1 proteins in both light and dark conditions, likely by repressing the expression of MAX2, an E3 ubiquitin ligase for BZR1.

Finally, on the basis of our data, we proposed a model to illustrate how light, in combination with 26S proteasome pathway and sugar levels, coordinate the homeostasis of BZR1 phosphorylation states and protein abundance (Figure 8). Briefly, fresh synthesized BZR1 protein might be in the inactive phosphorylated form and CHX treatment could block BZR1 biosynthesis. Dark induces the dephosphorylation while light promotes the phosphorylation of BZR1, mediated by PP2A and BIN2 respectively. dBZR1, the active and stable form of BZR1, can activate the expression of downstream target genes and mediate a series of BR responses, such as promoting cell elongation. On the other hand, pBZR1, the unstable form of BZR1, is easily degraded by the 26S proteasome pathway, mediated by various E3 ubiquitin ligases, such as MAX2 etc. Finally, sucrose could significantly promote the accumulation of total BZR1 protein in both light and dark conditions, likely by repressing the transcription of *MAX2*. In future studies, more experiments are required to further support this model, for instance, testing whether strigolactone application or MAX2 overexpression could suppress sucrose effects etc.

## Abbreviations

BES1: bri1-EMS-suppressor 1
bHLH: basic helix–loop–helix
BR: brassinosteroid
BRRE: BR-response element
BRZ: brassinazole
BZR1: brassinazole resistant 1
BRI1: BR insensitive 1
BIN2: BR insensitive 2
CHX: cycloheximide
COP1: constitutive photomorphogenesis 1
CT: cantharidin
ddH2O: doble distilled water
GSK3: glycogen synthase kinase 3
HY5: elongated hypocotyls 5
MAX2: more axillary growth locus 2
MS: Murashige and Skoog
PIF4: phytochrome interacting factor 4
PP2A: protein phosphatase 2A
qRT-PCR: real-time quantitative RT-PCR
RGA: repressor-of-ga1-3
